# Accelerated triacylglycerol production and altered fatty acid composition in oleaginous microalga *Neochloris oleoabundans* by overexpression of diacylglycerol acyltransferase 2

**DOI:** 10.1186/s12934-017-0677-x

**Published:** 2017-04-12

**Authors:** Paeka Klaitong, Sirirat Fa-aroonsawat, Wipa Chungjatupornchai

**Affiliations:** Institute of Molecular Biosciences, Mahidol University, Salaya Campus, Nakhon Pathom, 73170 Thailand

**Keywords:** Biofuel, Biodiesel, Diacylglycerol acyltransferase (DGAT), Microalgae, Lipids, Genetic engineering

## Abstract

**Background:**

Microalgae are promising sources of lipid triacylglycerol (TAG) for biodiesel production. However, to date, microalgal biodiesel is technically feasible, but not yet economically viable. Increasing TAG content and productivity are important to achieve economic viability of microalgal biodiesel. To increase TAG content, oleaginous microalga *Neochloris oleoabundans* was genetically engineered with an endogenous key enzyme diacylglycerol acyltransferase 2 (NeoDGAT2) responsible for TAG biosynthesis.

**Results:**

The integration of *NeoDGAT2* expression cassettes in *N. oleoabundans* transformant was confirmed by PCR. The neutral lipid accumulation in the transformant detected by Nile red staining was accelerated and 1.9-fold higher than in wild type; the lipid bodies in the transformant visualized under fluorescence microscope were also larger. The *NeoDGAT2* transcript was two-fold higher in the transformant than wild type. Remarkably higher lipid accumulation was found in the transformant than wild type: total lipid content increased 1.6-to 2.3-fold up to 74.5 ± 4.0% dry cell weight (DCW) and total lipid productivity increased 1.6- to 3.2-fold up to 14.6 ± 2.0 mg/L/day; while TAG content increased 1.8- to 3.2-fold up to 46.1 ± 1.6% DCW and TAG productivity increased 1.6- to 4.3-fold up to 8.9 ± 1.3 mg/L/day. A significantly altered fatty acid composition was detected in the transformant compared to wild type; the levels of saturated fatty acid C16:0 increased double to 49%, whereas C18:0 was reduced triple to 6%. Long-term stability was observed in the transformant continuously maintained in solid medium over 100 generations in a period of about 4 years.

**Conclusions:**

Our results demonstrate the increased TAG content and productivity in *N. oleoabundans* by *NeoDGAT2* overexpression that may offer the first step towards making microalgae an economically feasible source for biodiesel production. The strategy for genetically improved microalga presented in this study can be applied to other microalgal species possessing desired characteristics for industrial biofuel production.

## Background

Biofuels are thought to represent a secure, renewable and environmentally safe alternative to fossil fuels. Biodiesel, one of the commonly used biofuels, is predominantly produced from oleaginous plants. Because microalgae have much higher lipid and biomass productivity than terrestrial plants, can utilize saline or wastewater for their growth, and require non-arable land [1], they are promising sources of lipid triacylglycerol (TAG) for biodiesel production. To date, biodiesel production from microalgae is technically, but not yet economically, feasible. There are several challenges that need to be overcome before microalgal biodiesel can be economically produced at a commercial scale, one of which is the lack of microalgal strains with high TAG content and biomass [1, 2]. Increasing TAG content in microalgae possessing several desired characteristics could be achieved by targeted genetic engineering of the key genes in TAG biosynthesis pathway, offering the first step towards making microalgae an economically feasible source for biodiesel production [3–5]. To date, the microalgal oleaginous trait has been extensively studied primarily in the model green microalga *Chlamydomonas reinhardtii*, however, high genetic diversity for this trait has been demonstrated in microalgae [6].

The TAG biosynthetic pathway in microalgae is poorly known, however, it is considered to be most similar to that operating in higher plants [7]. In the de novo TAG biosynthetic pathway, diacylglycerol acyltransferase (DGAT; EC 2.3.1.20) catalyzing the final and committed step has been identified as the rate-limiting enzyme for lipid accumulation in plants [8, 9]. DGAT catalyzing the formation of TAG from diacylglycerol and Acyl-CoA is thought to be the key enzyme for de novo TAG biosynthesis in all organisms [7]. DGAT has also been suggested as one of the most promising target genes for genetic engineering to enhance TAG accumulation in microalgae [4]. Most microalgal species have been shown to have DGAT isozymes derived from one *DGAT* type 1 (*DGAT1*) and multiple *DGAT* type 2 (*DGAT2*) genes [7]. The isozymes DGAT1 and DGAT2 of *C. reinhardtii* have been predicted to localize in the chloroplast and endoplasmic reticulum, respectively [10]. DGAT2 has been identified as the potent enzyme in TAG biosynthesis [11–13].

Overexpression of *DGAT2* for enhancing TAG accumulation has been attempted so far in a few microalgal species with varying success. In *C. reinhardtii*, endogenous *DGAT2* overexpression neither boosts TAG accumulation nor alters the fatty acid composition [14], however, enhanced TAG accumulation has been observed when *DGAT2* expressed under a phosphorus-starvation inducible promoter [15]. Heterologous *DGAT2* expression has been shown to enhance neutral lipid accumulation but subsequently encounter gene silencing [16]. Enhanced lipid accumulation also has been observed in *Nannochloropsis oceanica* and *Phaeodactylum tricornutum* overexpressing endogenous *DGAT2* [17, 18], and *Scenedesmus obliquus* expressing heterologous *DGAT2* [19]. The maximum TAG content produced by *DGAT2*-overexpressing microalgae that has been reported so far is 11% of dry cell weight [15–17]. However, *DGAT2* overexpression has not been explored so far in oleaginous microalga *Neochloris oleoabundans*.

*Neochloris oleoabundans*, a taxonomic synonym of *Ettlia oleoabundans* [20], has been demonstrated to be one of the most suitable lipid sources for biodiesel production [21–23]. Under nitrogen starvation condition, *N. oleoabundans* produces 35–54% lipids of dry cell weight; up to 80% of its total lipids is TAG mainly comprised of the saturated fatty acids in the range of 16–20 carbons [24] ideal for biodiesel production. However, the knowledge concerning *N. oleoabundans* is very limited; no genomic sequences are available. To enable genetic manipulation of TAG biosynthesis and molecular genetics study, the cDNA encoding a functional DGAT2 protein of *N. oleoabundans* (*NeoDGAT2*) has been cloned [11] and the stable nuclear transformation system of *N. oleoabundans* has been established [25].

In this study, we tested whether overexpression of an endogenous key enzyme DGAT catalyzing the final step would affect lipid biosynthesis in oleaginous microalga. The *NeoDGAT2* expression cassettes were transformed into *N. oleoabundans.* The *NeoDGAT2*-overexpressing transformant was characterized in detail with regards to growth characteristics, neutral lipid accumulation and lipid bodies in the cells, lipid content and productivity, and fatty acid composition.

## Results

### Selection of *N. oleoabundans* transformants

**Fig. 1:**
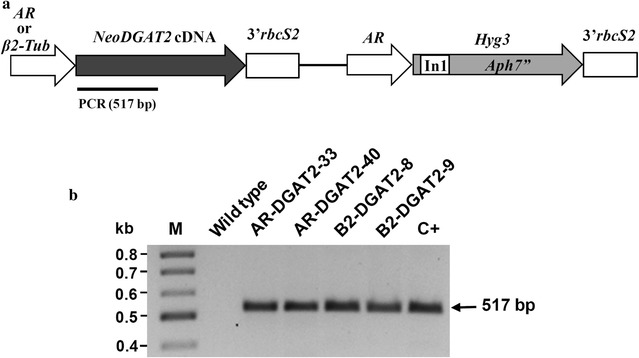
Generation of *N. oleoabundans* transformants. **a** Schematic diagram of plasmids pAR-DGAT2 and pB2-DGAT2 used to transform the *N. oleoabundans* cells. The *NeoDGAT2* cDNA (Accession no. GenBank: KJ470774) [11] was expressed by either promoter *AR* [28], or *β2*-*Tub* [29] and contained *3′rbcS2* [41] at 3′end. *Hyg3* gene used as selectable marker [40]. The 517-bp PCR amplicon, denoted by a *line*. **b** PCR confirmation of *NeoDGAT2*-expression cassette integration in the transformants. Genomic PCR of transformants AR-DGAT2, B2-DGAT2 and wild type was performed with primers specifically bind to *NeoDGAT2* coding sequence. The 517-bp amplicon was detected in the transformants but not in wild type which was used as negative control. *Lanes M*, 100-bp DNA ladder; C+, plasmid pAR-DGAT2 (used as positive control)

The unicellular microalga *N. oleoabundans* was transformed with plasmids pAR-DGAT2 and pB2-DGAT2 harboring endogenous diacylglycerol acyltransferase type 2 (*NeoDGAT2*) cDNA under the control of promoters *HSP70*-*RBCS2* (*AR*) and *β2*-*tubulin* (*β2*-*Tub*), respectively (Fig. 1a), via electroporation. The resulting transformants AR-DGAT2 and B2-DGAT2 were selected on hygromycin B-supplemented BBM agar with a transformation frequency of about 90 ± 10 colonies/1.5 × 10^7^ cells. To screen for clones with potential high neutral lipid accumulation, about 25 colonies selected from each plasmid transformation were grown on nitrogen-depleted (BBM-N) agar plates for 3 days and then stained with Nile red, a reagent that yields brilliant fluorescence in a neutral lipid environment [26]. On the basis of high Nile red fluorescence intensity, transformants AR-DGAT2-33, AR-DGAT2-40, B2-DGAT2-8 and B2-DGAT2-9 were selected for subsequent experiments.

### Evaluation of *NeoDGAT2*-expression cassette integration

The integration of *NeoDGAT2* expression cassettes in *N. oleoabundans* was confirmed by genomic PCR using primer pair specific to *NeoDGAT2* coding sequence. The expected amplicon of 517 bp was detected in the four selected transformants AR-DGAT2-33, AR-DGAT2-40, B2-DGAT2-8 and B2-DGAT2-9, but not in wild type (Fig. 1b). The 517-bp amplicon was subjected to DNA sequencing and confirmed to be *NeoDGAT2* coding sequence. Thus, the *NeoDGAT2*-expression cassettes were successfully introduced into the transformants. Partial sequences from genomic walking revealed that the amplicon from the resident *NeoDGAT2* gene including introns was at least 3 kb. Because the PCR condition used in this study was designed to amplify amplicon of about 1 kb, the amplicon from the resident *NeoDGAT2* gene was not amplified. The PCR-positive transformants were further analyzed for growth characteristics.

### Growth of transformants

**Fig. 2:**
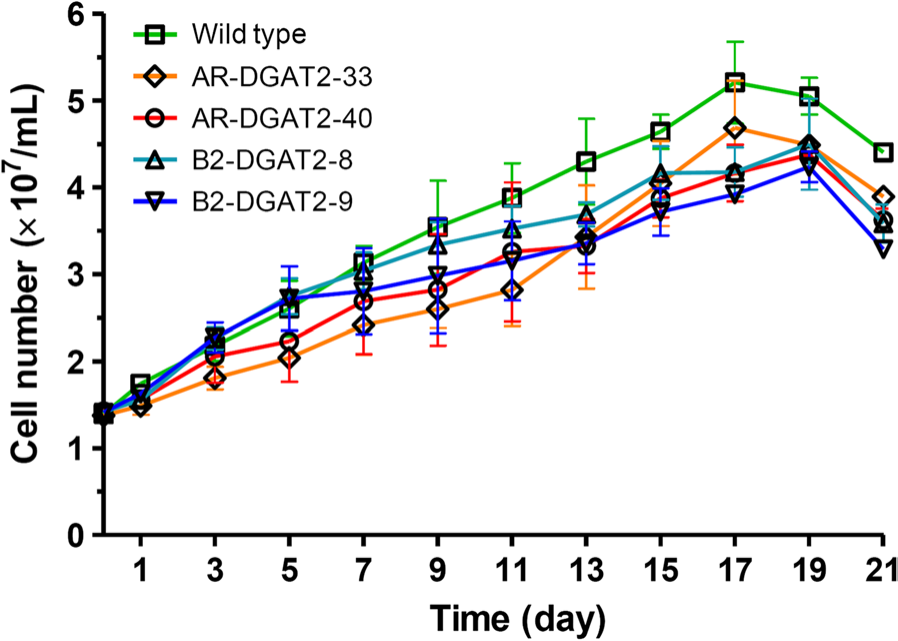
Growth curve of *N. oleoabundans* transformants AR-DGAT2 and B2-DGAT2 during N-sufficient growth condition. Each value represents mean ± SD (n = 3)

To evaluate whether *NeoDGAT2* overexpression had any effect on growth characteristics, we analyzed growth curve of the transformants and wild type under N-sufficient growth condition. All of the selected transformants showed overall similar growth curve compared to wild type, while slightly lower growth during the stationary phase (Fig. 2). However, the doubling time during exponential growth of transformants AR-DGAT2-40 (6.8 ± 1.0 days), B2-DGAT2-8 (7.0 ± 1.0 days), and B2-DGAT2-9 (7.3 ± 1.3 days), except AR-DGAT2-33 (9.7 ± 0.9 days), was not significantly different from that of wild type (6.4 ± 0.4 days) at *p* < 0.01. Thus, *NeoDGAT2* overexpression did not have an apparent effect on the growth of transformants AR-DGAT2-40, B2-DGAT2-8, and B2-DGAT2-9.

### Neutral lipid analysis by Nile red staining

**Fig. 3:**
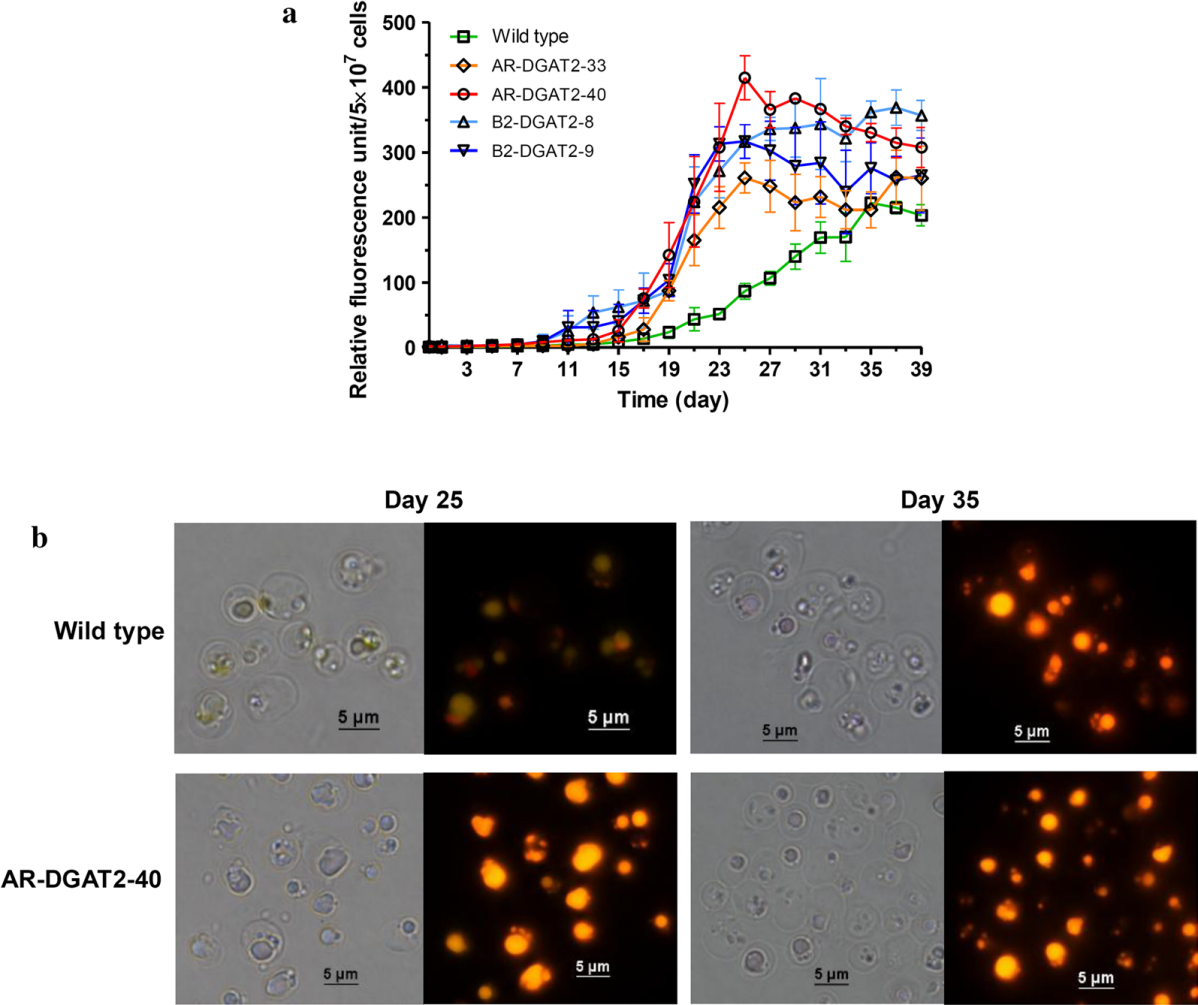
Detection of neutral lipid in the transformants using Nile red staining. **a** Relative Nile red fluorescence of transformants AR-DGAT2, B2-DGAT2 and wild type during N-starvation growth condition I. All transformants were found to reach maximum Nile red fluorescence earlier than wild type. Each value represents mean ± SD (n = 3). **b** Lipid bodies in transformant AR-DGAT2-40 and wild type (at indicated time point) visualized under a bright field microscope (*left panel*) and fluorescence microscope (*right panel).* Intense golden-color lipid bodies observed in transformant AR-DGAT2-40, light golden-color lipid bodies in wild type

*Neochloris oleoabundans*, like many microalgae, accumulates neutral lipids under N-starvation condition [22, 24, 27]. To evaluate the lipid-production potential of the transformants, neutral lipids in the cells cultured under N-starvation condition I (see “Methods”) were stained with fluorescent dye, Nile red. The neutral lipid accumulation in the transformants AR-DGAT2-33, AR-DGAT2-40, B2-DGAT2-8, B2-DGAT2-9 was accelerated and dramatically increased; all transformants were found to reach maximum neutral lipid content (day 25) earlier than wild type (day 35) (Fig. 3a). Among the transformants, AR-DGAT2-40 showed the highest neutral lipid content which increased to 1.9-fold compared to the maximum content in wild type. Therefore, transformant AR-DGAT2-40 was selected for subsequent experiments.

The lipid bodies in transformant AR-DGAT2-40 and wild type were further visualized under fluorescence microscope. Intense golden-color lipid bodies with larger volume were observed in transformant AR-DGAT2-40, whereas, light golden-color lipid bodies with smaller volume in wild type. The red-color background was due to chlorophyll autofluorescence (Fig. 3b). Thus, *NeoDGAT2* overexpression in transformant AR-DGAT2-40 enhanced the accumulation of lipid bodies that was in accordance with the increased neutral lipid content (Fig. 3a).

### Evaluation of *NeoDGAT2* transcript

**Fig. 4:**
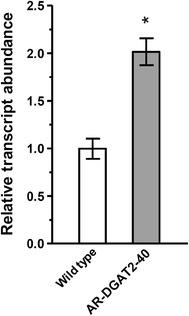
Relative *NeoDGAT2* transcript abundance in transformant AR-DGAT2-40. The levels of *NeoDGAT2* transcript during N-starvation growth condition I were determined by quantitative real-time PCR. The values are normalized to the expression level of endogenous *NeoActin.* Each value represents mean ± SD (n = 3). Significant difference between transformant AR-DGAT2-40 and wild type is indicated (*p < 0.01, *t* test)

To examine whether the integrated *NeoDGAT2*-expression cassette in the transformant expressed at transcriptional level, the relative *NeoDGAT2* transcript abundance in cells cultured under N-starvation condition was determined by quantitative real-time PCR (qPCR) using *NeoActin* transcript as a reference. Transformant AR-DGAT2-40 was observed to have *NeoDGAT2* transcript increased twofold compared to wild type (Fig. 4), indicating that the increased transcript was enhanced by *NeoDGAT2* overexpression.

### Lipid productivity analysis

For economically viable to produce biodiesel, microalgal strains should have both high lipid content and enhanced biomass. Lipid analysis in this study was performed in *N. oleoabundans* cultured under N-starvation condition I and the enhanced cell growth by increasing aeration, N-starvation conditions II. The cells were harvested when they reached maximum neutral lipid accumulation monitored by Nile red staining and the dried cells were determined gravimetrically as dry cell weight (DCW). Biomass of transformant AR-DGAT2-40 under N-starvation condition I and II were 117 ± 12 and 449 ± 57 mg DCW/L, respectively, increasing about 3.8-fold; while that of wild type were 163 ± 21 and 462 ± 27 mg DCW/L, respectively, increasing about 2.8-fold. Under the same growth condition, biomass was not significantly different between the transformant and wild type (*p* < 0.05). Thus, the biomass was enhanced by increasing aeration under N-starvation condition and not affected by *NeoDGAT2* overexpression.

**Fig. 5:**
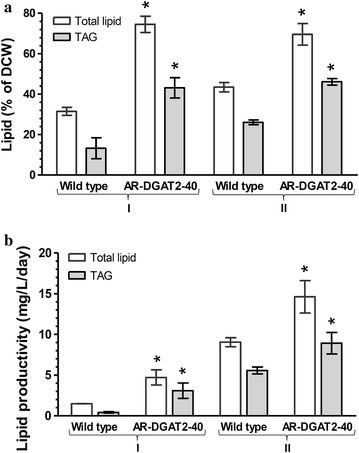
Lipid analysis of transformant AR-DGAT2-40. **a** Lipid content and **b** Lipid productivity of wild type and transformant AR-DGAT2-40. Lipids extracted from cells grown under N-starvation condition I and II. N-starvation condition II for increasing cell mass was the same as condition I, except increasing aeration. Each value represents mean ± SD (n = 3). Significant difference between transformant AR-DGAT2-40 and wild type in the same growth condition is indicated (*p < 0.01, *t* test)

Because TAG is the major component for biodiesel production, the TAG content separated from total lipids using TLC was quantified. Up to 66% of total lipids extracted from transformant AR-DGAT2-40 and wild type were TAG (Fig. 5a). The lipid content and productivity in transformant AR-DGAT2-40 were compared to wild type: under N-starvation condition I, total lipid content in transformant (74.5 ± 4.0% DCW) increased 2.3-fold, TAG content (43.1 ± 5.0% DCW) increased 3.2-fold, total lipid productivity (4.7 ± 0.9 mg/L/day) increased 3.2-fold, TAG productivity (3.1 ± 0.9 mg/L/day) increased 4.3-fold; under N-starvation condition II, total lipid content in transformant (69.6 ± 5.3% DCW) increased 1.6-fold, TAG content (46.1 ± 1.6% DCW) increased 1.8-fold, total lipid productivity (14.6 ± 2.0 mg/L/day) increased 1.6-fold and TAG productivity (8.9 ± 1.3 mg/L/day) increased 1.6-fold (Fig. 5a, b). Therefore, transformant AR-DGAT2-40 was observed to have dramatically increase in lipid accumulation both under N-starvation condition I and II when compared to wild type: the total lipid content and productivity increased 1.6-to 2.3-fold and 1.6- to 3.2-fold, respectively; while the TAG content and productivity increased 1.8- to 3.2-fold and 1.6- to 4.3-fold, respectively (Fig. 5a, b). *NeoDGAT2* overexpression markedly improved TAG content and productivity in the microalga.

### Fatty acid composition analysis

**Fig. 6:**
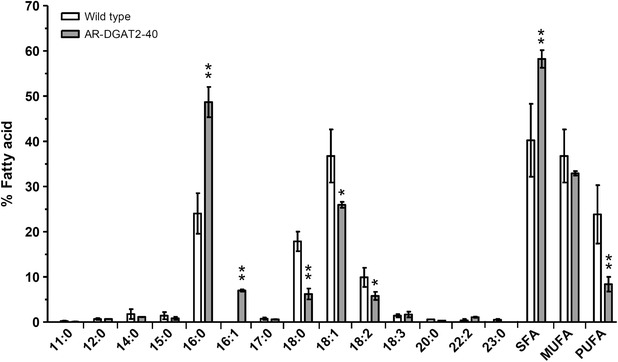
Fatty acid composition in transformant AR-DGAT2-40. Fatty acid methyl esters in transformant AR-DGAT2-40 and wild type were analyzed using GC-FID. Fatty acid composition in the predominant region of carbon chain length C16-C18 in transformant AR-DGAT2-40 was altered when compared to wild type: C16:0 and C16:1 increased, whereas C18:0, C18:1 and C18:2 were reduced. SFA, MUFA and PUFA are saturated, monounsaturated and polyunsaturated fatty acids. Each value represents mean ± SD (n = 3). Significant difference between transformant AR-DGAT2-40 and wild type is indicated (**p < 0.01, *p < 0.02, *t* test)

Because fatty acid (FA) composition can impact the quality of the biodiesel, we tested whether *NeoDGAT2* overexpression would have any effect on FA composition. Fatty acid methyl esters (FAME) obtained by transesterification of TAG were analyzed using gas chromatography equipped with flame ionization detector (GC-FID). FAME in the chain-length range of C11–C23 was determined by comparison with the standard reference. The palmitic acid (C16:0), a saturated fatty acid (SFA) and oleic acid (C18:1), a monounsaturated fatty acid (MUFA), were the most abundant FA in the TAG (Fig. 6). The FA of C16 increased whereas C18 was reduced in transformant AR-DGAT2-40 compared to wild type: C16:0 increased double the amount to 49% from 24% and C16:1 (palmitoleic acid, MUFA) increased to 7% from 0%, whereas C18:0 (stearic acid, SFA) was reduced triple to 6% from 18%, C18:1–26% from 37%, and C18:2 (Linoleic acid, polyunsaturated fatty acid (PUFA)) to 6% from 10%. The overall SFA increased to 58% from 40%, MUFA was not significantly different from the wild type, whereas PUFA was reduced to 8% from 24% (Fig. 6). The C16:0 increased double, suggesting that C16:0-acyl-CoA might be a preferred substrate of NeoDGAT2. A significant alteration of the FA composition in transformant AR-DGAT2-40 was due to *NeoDGAT2* overexpression.

### Long-term stability of transformants

Transformants AR-DGAT2 and B2-DGAT2 were continuously maintained in solid BBM medium by subculturing (every 2 weeks) over 100 generations in a period of about 4 years. The transformants were periodically checked for neutral lipid accumulation by Nile red staining; no loss in higher lipid-accumulation than wild-type trait was observed in all the transformants used in this study, indicating the *NeoDGAT2* overexpression stability.

## Discussion

This study is based on the overexpression of endogenous diacylglycerol acyltransferase type 2 (*NeoDGAT2*) in *N. oleoabundans* to improve triacylglycerol (TAG) accumulation for potential biodiesel production. The important prerequisites for *NeoDGAT2* overexpression in *N. oleoabundans* are the availabilities of: (i) the stable nuclear transformation system [25], (ii) the *NeoDGAT2* cDNA encoding a functional DGAT protein. NeoDGAT2 fused with His tag at the C-terminus for facilitating Western blot analysis has been shown to reduce the NeoDGAT2 activity [11]. NeoDGAT2 without tag was therefore used in this study, and (iii) the functional promoter that can drive the expression of the *NeoDGAT2.* Because no data concerning *N. oleoabundans* endogenous promoter are available, promoters *AR* and *β2*-*Tub* from *C. reinhardtii* [28, 29] that have been shown to function in *N. oleoabundans* with similar activity [25] were utilized in this study. Among the transformants, AR-DGAT2-40 showed the highest neutral lipid content which increased to 1.9-fold compared to the maximum content in wild type (Fig. 3a). This transformant overexpressing *NeoDGAT2* under *AR* promoter may produce more lipids at high temperature, because *AR* promoter has been shown to be heat inducible in *N. oleoabundans* [25]. However, cells cultured under N-starvation condition already have poor growth [22], additional stress conditions were omitted in this study.

The lipid bodies with higher fluorescence intensities and larger volume were observed in transformant AR-DGAT2-40 than in wild type (Fig. 3b). The lipid bodies under N starvation in green microalgae has been shown as a results of increased de novo synthesis of TAG [6]. Lipid analysis in this study was performed in cells cultured under N-starvation condition I and the enhanced cell growth by increasing aeration, N-starvation conditions II. Biomass of transformant AR-DGAT2-40 under N-starvation condition II was about 3.8-fold higher than under condition I. Thus, the biomass was enhanced by increasing aeration under N-starvation condition and not affected by *NeoDGAT2* overexpression. The TAG productivity in the transformant could be further improved by optimizing the growth condition to enhance biomass. The TAG content of 46.1 ± 1.6% DCW produced by the transformant in this study (Fig. 5a) was the highest in comparison to those produced by *DGAT2*-overexpressing microalgae reported so far with maximum TAG content of 11% DCW [15–17].

A significant alteration of the FA composition was observed in transformant AR-DGAT2-40 compared to wild type. The C16:0 in the transformant increased double, suggesting that C16:0-acyl-CoA might be a preferred substrate of NeoDGAT2; in agreement with preferred substrate of *C. reinhardttii* DGAT2 [30]. Similar incident of FA composition alteration has been reported previously. Expression of *Brassica DGAT2* in *C. reinhardtii* increases PUFA while reduces SFA [16]. Overexpression of *DGAT2* in *N. oceanica* increases SFA and PUFA whereas decreases MUFA [17]. FA compositions of TAG in *DGAT2*-overexpressing *C. reinhardtii* are different between N- and phosphorus-limited growth conditions [15].

The FA composition of TAG plays an important role in some critical parameters of the biodiesel, such as cetane number [31]. Cetane number is one of the important factors in determining the quality of diesel fuel; the higher number the more easily the fuel will combust in a compression setting [32]. High cetane numbers have been observed for esters of SFA such as C16:0 and C18:0 and MUFA such as C18:1 [33]. However, increased PUFA content unfavorably impacts the cetane number and oxidation stability of the biodiesel [31]. The FA composition of *N. oleoabundans* TAG consists of mainly C18:1 followed by C16:0 and C18:0 [22] (Fig. 6). Linolenic acid (C18:3) proportion was below 12% which meets the requirements of the European Standard EN 14214 for biodiesel production [22, 34] (Fig. 6). In this study, SFA C16:0 of transformant AR-DGAT2-40 increased double to 49%, PUFA was reduced triple to 8% (Fig. 6), thus the altered FA composition will still maintain the good quality of *N. oleoabundans* biodiesel production.

Lipid quality in most of the microalgal species is not good enough to be directly used as a substrate for producing good quality biodiesel [16]. However, *N. oleoabundans* has been demonstrated to have the best performance potential for biodiesel production, i.e. growth characteristics, lipid content, fatty acid composition, acid value and iodine value that meets the requirements of the European Standard EN 14214 for biodiesel production [22, 34]. Therefore, *N. oleoabundans* is a suitable renewable lipid source for biodiesel production.

Silencing of a foreign gene shortly after nuclear integration is not uncommon and often permanent [35, 36]. *C. reinhardtii* has been found to often silence or down regulate the non-required heterologous genes when expressed at high levels; the losses of trait or genetic changes in *C. reinhardtii* cultures have been observed in solid medium [16, 37]. Similar phenomenon of heterologous gene silencing has been observed in *N. oleoabundans*; the green fluorescent protein (GFP) activity in the transformants introduced with *Gfp* gene [25] seemed to diminish when the transformants continuously maintained in solid BBM medium for over a year (Kitraksa and Chungjatupornchai, unpublished observations). To avoid heterologous gene silencing, in the present study, endogenous *NeoDGAT2* was transformed into *N. oleoabundans* for enhancing TAG accumulation. Transformants AR-DGAT2 and B2-DGAT2 were continuously maintained in solid BBM medium for a long time; no loss in higher lipid-accumulation than wild-type trait was observed for over 100 generations in a period of about 4 years, indicating the *NeoDGAT2* overexpression stability. Endogenous *DGAT2* overexpression in *C. reinhardtii* has been shown to neither boost TAG accumulation nor alter the FA composition despite the higher levels of transcripts observed that might be due to unknown negative feedback inhibition [14]. However, such negative feedback inhibition was not observed in *N. oleoabundans*; *NeoDGAT2* overexpression altered FA composition and markedly increased TAG production (Figs. 5, 6).

## Conclusions

We successfully generated *N. oleoabundans* transformant overexpressing NeoDGAT2 with remarkably accelerated and higher TAG content and productivity. The TAG productivity in the transformant could be further improved by optimizing the growth condition to enhance biomass. A significantly altered fatty acid composition was detected in the transformant compared to wild type. Long-term stability was observed in the transformant continuously maintained in solid medium over 100 generations in a period of about 4 years. Thus, the increasing TAG content in *N. oleoabundans,* one of the most suitable lipid sources for biodiesel production, was achieved by targeted genetic engineering of the key enzyme in TAG synthesis pathway, DGAT2. This may offer the first step towards making microalgae an economically feasible source for biodiesel production. The strategy for genetically improved microalga presented in this study can be applied to other microalgal species possessing desired characteristics for industrial biofuel production.

## Methods

### Strain and growth conditions

*Neochloris oleoabundans* strain UTEX 1185, obtained from the Algal Culture Collection at the University of Texas, was cultured in liquid or in solid (1.5% Difco Bacto agar) Bold’s basal medium (BBM) [38, 39] at 30 °C under constant illumination of 55–60 μmol photons/m^2^/s. Cultures in liquid medium were inoculated with cells at starting density of ~1.5 × 10^7^ cells/mL (OD_750_ = 0.3). For cell growth under nitrogen (N)-sufficient condition, cultures were maintained in 500 mL Erlenmeyer flasks containing 150 mL of BBM; the flasks were sealed and shaken at 100 rpm. Cell concentration of the cultures was monitored using spectrophotometer for optical density measurement at 750 nm and using hemocytometer for cell counting. Doubling time of the cells was calculated as described [22] using the formula *t*_d_ = ln 2/µ_max_, where µ_max_ is the maximum specific growth rate, calculated as the maximum slope from the plot of ln OD versus culture time. For N-starvation condition I, the cells grown in BBM at exponential phase were harvested, washed and resuspended in 150 mL of BBM without NaNO_3_ (BBM-N) in 500 mL Erlenmeyer flasks supplied with 12 L/h bubbling-filtered air. N-starvation condition II for enhancing growth was the same as condition I, except cells were resuspended in 200 mL of BBM-N, shaken at 50 rpm and supplied with 50 L/h bubbling-filtered air.

### Construction of transformation vectors

To construct *NeoDGAT2* cDNA (GenBank: KJ470774) under the control of *HSP70*-*RBCS2* (*AR*) promoter of *C. reinhardtii* [28] and *β2*-*tubulin* (*β2*-*Tub*) promoter of *C. reinhardtii* [29], plasmids pAR-DGAT2 and pB2-DGAT2 harboring the gene cassettes *AR*-*NeoDGAT2*-*3′rbcS2* and (*β2*-*Tub)*-*NeoDGAT2*-*3′rbcS2*, respectively (Fig. 1a), were constructed by replacing the *AR*-*ChGfp*-*3′rbcS2* fragment of pChGFP-Hyg3 [25] with the PCR fragments containing: (i) *AR* promoter from pCB740 [28] or *β2*-*Tub* promoter from pHyg3 [40], (ii) *NeoDGAT2* cDNA (GenBank: KJ470774) from pGEM-NeoDGAT2 [11], and (iii) 3′UTR of *3′rbcS2* from pCrGFP [41]. Both pAR-DGAT2 and pB2-DGAT2 harbored selectable marker gene *Hyg3* conferring hygromycin B resistance [25, 40].

### Transformation of *N. oleoabundans*

To generate transformants overexpressing DGAT2, plasmids pAR-DGAT2 and pB2-DGAT2 were transformed into the *N. oleoabundans* nuclear genome using electroporation as described [25]. *N. oleoabundans* cells were electroporated using a Gene Pulser (Bio-Rad Labs.) set resistance at 200 Ω, capacitance at 25 μF and electric field strength at 1000 V/cm. The electroporated cells were spread on a BBM agar plate containing 5 μg/mL hygromycin B. The resulting transformants AR-DGAT2 and B2-DGAT2 appeared after incubation for 2 weeks.

### Genomic PCR analysis

The *NeoDGAT2*-expression cassettes integrated into the nuclear genome of *N. oleoabundans* were verified using genomic PCR. The genomic DNA of *N. oleoabundans* was isolated as described [42]. Genomic PCR was performed with primers DGAT2-F2 (CGGGATCCTAGCTAGCATGGCGGCTCAGCGCGGTTTCG) and DGAT2-R2 (CCACAGACCTGCCCTTCTTCAGC) specifically bind to *NeoDGAT2* coding sequence. The touchdown PCR was carried out 11 cycles for the first phase (denature for 10 s at 98 °C, annealing 30 s at 80 °C which was reduced by 1 °C every successive cycle until 70 °C, extension 30 s at 72 °C) and 25 cycles for second phase (denature for 10 s at 98 °C, annealing 30 s at 70 °C, extension 30 s at 72 °C), including initial denaturation for 3 min at 98 °C and final extension for 7 min at 72 °C. PCR product was examined in a 1.5% agarose gel and further confirmed by DNA sequencing analysis. The amplicon of the *NeoDGAT2* coding sequence was 517 bp.

### Nile red fluorescence assay and microscopy

To evaluate the level of neutral lipids, *N. oleoabundans* grown under N-starvation condition I (~1.5 × 10^7^ cells/mL) was stained with fluorescent dye Nile red dissolved in acetone to final concentration of 1 µg/mL and incubated in the dark for 10 min. The fluorescence intensity was measured in a 96-well plate using a spectrofluorometer (Beckman Coulter DTX-880, USA) with excitation at 535 nm and emission at 574 nm. The observed intensities were corrected by subtracting the fluorescence value difference in Nile red stained and unstained cells. Specific fluorescence intensities were normalized by cell numbers. The lipid bodies in the cells stained with Nile red were visualized under an inverted fluorescence microscope (Nikon Eclipse Ti-S, Japan) with excitation at 420–490 nm and emission at 520 nm.

### Quantitative real time PCR analysis

Relative *NeoDGAT2* transcript abundance was quantified using quantitative real-time PCR (qPCR). Total RNA was extracted from cells cultured under N-starvation condition I at stationary phase using TRI Solution (GeneMark, Taiwan). The cDNA was prepared from the total RNA using oligo (dT)18 primer and RevertAid H Minus First Strand cDNA Synthesis Kit (Thermo Scientific, Canada). The cDNA was amplified by KAPA SYBR FAST qPCR Kit (Kababiosystems, USA) using *NeoDGAT2*-gene specific primers: DGAT-RT-F1 (GGCGACAAAGGTCTTCCTCC) and DGAT-RT-R1 (GGCTCGTATCCGATTACAAAGG) and endogenous *Actin* (*NeoActin*)-gene specific primers: NeoActin-F1 (ACACTGTGCCCATCTATGAGGG) and NeoActin-R1 (CTTGATGTCACGCACGATTTCG). Mastercycler realplex4 and realplex software (Eppendorf, Germany) were used for the analysis. Fold difference of transcript was calculated using the ΔΔCt method. The *NeoDGAT2* transcript level was normalized to *NeoActin* transcript used as a reference.

### Lipid extraction and quantification

Total lipids of *N. oleoabundans* grown under N-starvation condition were extracted based on Bligh and Dyer method [43]. The cell pellet of 30 OD_750_ (≈50 mg) was suspended in chloroform:methanol (2:1, v/v). The cells were lysed using 0.5 mm glass beads with vortexing at 2700 rpm (Vortex Genie2 G560E, Scientific Industries, USA), then chloroform:water (1:1, v/v) was added to the mixture. Chloroform phase was collected and evaporated using nitrogen gas. Total lipids were then determined gravimetrically. TAG was subsequently separated from total lipids by thin-layer chromatography (TLC) using the solvent system hexane:diethyl ether:acetic acid (70:30:1, v/v/v) and glyceryl trioleate (92860 Sigma-Aldrich, USA) as a reference substance. Quantification of TAG stained with iodine was performed using Quantity One 1-D analysis software (Bio-Rad Labs., USA). Dry cell weight (DCW) of a sample was determined gravimetrically after drying *N. oleoabundans* cells. Total lipid and TAG content was calculated as percentage of dry cell weight (% DCW). The lipid productivity was calculated using the formula

$$ \begin{aligned} P_{\text{Lipid}} \left( {{\text{mg}}/{\text{L}}/{\text{day}}} \right) &= [C_{\text{Lipid}} \left( {{\text{mg}}/{\text{mg}}} \right) \\&\quad\times {\text{DCW }}\left( {{\text{mg}}/{\text{L}}} \right)]/{\text{Time }}\left( {\text{day}} \right),\end{aligned} $$

where *C*_Lipid_ is lipid content of cells, DCW is dry cell weight, and Time is the cultivation period, as described [21].

### Fatty acid composition analysis

To obtain fatty acid methyl esters (FAME), TAG extracted from TLC was incubated with 5% (v/v) sulfuric acid in methanol at 70 °C for 3 h in the presence of glyceryl trinonadecanoate (91988 Sigma-Aldrich) used as internal standard. FAME analysis was carried out using gas chromatography-flame ionization detector (GC-FID) (7890A GC system, Agilent USA) equipped with Agilent DB-WAX capillary column (30 m × 0.25 mm × 0.25 µm) and helium as the carrier gas. The oven temperature was increased from 50 to 200 °C at a rate of 28.5 °C/min, 200 to 240 °C at a rate of 3.4 °C/min and held at 240 °C for 16 min. Supelco 37-component FAME mix (Supelco 47885-U, Sigma-Aldrich) was used as the external standard to identify retention time for specific FAME. Fatty acid composition was calculated as percentage of the total fatty acids present in the sample, determined from the peak areas.

### Statistical analysis

To determine the statistical differences between wild type (control) and transformant samples, two-tailed student’s *t* test was performed using SPSS Base 16.0 software (SPSS, USA).

## Abbreviations

DCW: dry cell weight
DGAT: diacylglycerol acyltransferase
GC-FID: gas chromatography-flame ionization detector
N: nitrogen
qPCR: quantitative real-time PCR
FA: fatty acids
FAME: fatty acid methyl ester
MUFA: monounsaturated fatty acid
PUFA: polyunsaturated fatty acid
SFA: saturated fatty acid
TAG: triacylglycerol
TLC: thin-layer chromatography
